# Efficacy and safety of secukinumab administration by autoinjector in patients with psoriatic arthritis: results from a randomized, placebo-controlled trial (FUTURE 3)

**DOI:** 10.1186/s13075-018-1551-x

**Published:** 2018-03-15

**Authors:** Peter Nash, Philip J. Mease, Iain B. McInnes, Proton Rahman, Christopher T. Ritchlin, Ricardo Blanco, Eva Dokoupilova, Mats Andersson, Radhika Kajekar, Shephard Mpofu, Luminita Pricop, on behalf of the FUTURE 3 study group

**Affiliations:** 1 Department of Medicine, University of Queensland, Brisbane, Australia; 2 Swedish Medical Centre and University of Washington, Seattle, WA USA; 3 University of Glasgow, Glasgow, UK; 4 Memorial University of Newfoundland, St. John’s, NL Canada; 5 Allergy/Immunology and Rheumatology Division, University of Rochester, Rochester, NY USA; 6 Hospital Universitario Marqués de Valdecilla, IDIVAL, Santander, Spain; 7 Medical Plus, s.r.o., Uherské Hradiště, and University of Veterinary and Pharmaceutical Sciences, Faculty of Pharmacy, Brno, Czech Republic; 8 Novartis Pharma AG, Basel, Switzerland; 9 Novartis Pharmaceuticals Corporation, East Hanover, NJ USA

**Keywords:** Psoriatic arthritis, Secukinumab, Autoinjector, Interleukin-17a, FUTURE 3 study

## Abstract

**Background:**

The study aimed to assess 52-week efficacy and safety of secukinumab self-administration by autoinjector in patients with active psoriatic arthritis (PsA) in the FUTURE 3 study (ClinicalTrials.gov NCT01989468).

**Methods:**

Patients (≥ 18 years of age; *N* = 414) with active PsA were randomized 1:1:1 to subcutaneous (s.c.) secukinumab 300 mg, 150 mg, or placebo at baseline, weeks 1, 2, 3, and 4, and every 4 weeks thereafter. Per clinical response, placebo-treated patients were re-randomized to s.c. secukinumab 300 or 150 mg at week 16 (nonresponders) or week 24 (responders) and stratified at randomization by prior anti-tumor necrosis factor (TNF) therapy (anti-TNF-naïve, 68.1%; intolerant/inadequate response (anti-TNF-IR), 31.9%). The primary endpoint was the proportion of patients achieving at least 20% improvement in American College of Rheumatology response criteria (ACR20) at week 24. Autoinjector usability was evaluated by Self-Injection Assessment Questionnaire (SIAQ).

**Results:**

Overall, 92.1% (300 mg), 91.3% (150 mg), and 93.4% (placebo) of patients completed 24 weeks, and 84.9% (300 mg) and 79.7% (150 mg) completed 52 weeks. In the overall population (combined anti-TNF-naïve and anti-TNF-IR), ACR20 response rate at week 24 was significantly higher in secukinumab groups (300 mg, 48.2% (*p* < 0.0001); 150 mg, 42% (*p* < 0.0001); placebo, 16.1%) and was sustained through 52 weeks. SIAQ results showed that more than 93% of patients were satisfied/very satisfied with autoinjector usage. Secukinumab was well tolerated with no new or unexpected safety signals reported.

**Conclusions:**

Secukinumab provided sustained improvements in signs and symptoms in active PsA patients through 52 weeks. High acceptability of autoinjector was observed. The safety profile was consistent with that reported previously.

**Trial registration:**

ClinicalTrials.gov NCT01989468. Registered 21 November 2013.

EudraCT 2013–004002-25. Registered 17 December 2013.

**Electronic supplementary material:**

The online version of this article (10.1186/s13075-018-1551-x) contains supplementary material, which is available to authorized users.

## Background

Psoriatic arthritis (PsA) is an immune-mediated chronic inflammatory disease that affects peripheral and axial joints, entheses, and skin and is associated with impaired physical function and poor quality of life [1–3].

Nonsteroidal anti-inflammatory drugs (NSAIDs) and nonbiologic disease-modifying anti-rheumatic drugs (DMARDs) are used as first-line therapy for PsA; however, these often only partially control disease symptoms [4].

Introduction of tumor necrosis factor (TNF)-blocking therapy improved the treatment of patients with PsA [5–7]. However, relapse or loss of response to anti-TNF therapy and adverse events (AEs) are common, highlighting an unmet clinical need for improved disease control [8, 9].

The interleukin-17A (IL-17A) pathway is proposed to play a key role in the pathogenesis of PsA [10].

Secukinumab, a fully human monoclonal antibody that selectively neutralizes IL-17A, has shown significant efficacy in the treatment of moderate to severe psoriasis and PsA, with a rapid onset of action, sustained responses, and a favorable safety profile [6, 11–14].

Placebo-controlled, double-blind, phase III trials with secukinumab (FUTURE 1 and FUTURE 2) have reported significant improvements in key clinical domains of PsA sustained through week 104 in FUTURE 2 and week 156 in FUTURE 1 [6, 15–19].

Here we report the primary efficacy and safety results over a 52-week period from the FUTURE 3 study (ClinicalTrials.gov NCT01989468), which involves subcutaneous (s.c.) self-administration of secukinumab via autoinjector in patients with active PsA.

## Methods

### Patients

Patients included were ≥ 18 years of age, meeting the ClASsification criteria for Psoriatic ARthritis (CASPAR) and having active disease, defined as at least three tender joints out of 78 and at least three swollen joints out of 76, despite previous treatment with NSAIDs, DMARDS, or anti-TNF agents. Patients treated with up to three anti-TNF agents could enroll if they had an inadequate response for at least 3 months or had stopped anti-TNF therapy due to safety/tolerability reasons (anti-TNF-IR), and had an appropriate washout period before randomization.

Concomitant use of oral corticosteroids (≤ 10 mg/day prednisone or equivalent) and methotrexate (MTX; ≤ 25 mg/week) was allowed if the dose was stable for at least 2 weeks and 4 weeks before randomization, respectively.

The exclusion criteria were: previous use of any biological agent other than anti-TNF agents or the use of > 3 anti-TNF agents; active inflammatory diseases other than PsA; active infection in the 2 weeks before randomization, or a history of ongoing, chronic, or recurrent infections, or evidence of tuberculosis infection; history of malignant disease within the past 5 years (excluding basal cell carcinoma or actinic keratosis, in-situ cervical cancer, or noninvasive malignant colon polyps); and pregnancy.

### Study design

FUTURE 3 is an ongoing, multicenter, randomized, double-blind, double-dummy, placebo-controlled, parallel-group, 3-year study assessing the use of s.c. secukinumab self-administration with an autoinjector in patients with active PsA.

The study is being conducted at 74 centers across Australia, Bulgaria, Canada, Czech Republic, Germany, Italy, the Netherlands, Russia, Spain, Switzerland, the United Kingdom, and the USA. Following a 10-week screening period, eligible patients were randomized (1:1:1) by means of an interactive response technology (IRT) to one of two secukinumab dose groups (secukinumab 300 mg or 150 mg) or placebo.

Patients in the secukinumab groups received s.c. secukinumab at a dose of 300 mg (2 × 1.0 ml autoinjector) or 150 mg (1.0 ml autoinjector + 1.0 ml placebo autoinjector) at baseline, weeks 1, 2, 3, and 4, and every 4 weeks thereafter. Patients in the placebo group (2 × 1.0 ml placebo autoinjector) were treated according to the same administration schedule as the active drug.

Patients were stratified at randomization based on previous anti-TNF therapy use as anti-TNF-naïve or anti-TNF-IR; at least 60% of patients in each treatment arm (secukinumab 300 mg, secukinumab 150 mg, and placebo) were anti-TNF-naïve.

At week 16, patients were classified either as responders (≥ 20% improvement from baseline in both tender joint count (TJC) and swollen joint count (SJC)) or nonresponders. Placebo patients were re-randomized to receive secukinumab 300 or 150 mg s.c. every 4 weeks at week 16 (nonresponders) or week 24 (responders; Additional file 1: Figure S1).

The study was conducted in accordance with the principles of the Declaration of Helsinki. All centers received approval from independent ethics committees (IECs) or institutional review boards (IRBs). The names of IECs or IRBs that approved the study and the associated approval numbers where applicable are presented in Additional file 1: Table S1. Patients provided written informed consent before starting the study-related procedures.

### Outcomes

The primary efficacy endpoint was the proportion of patients achieving an American College of Rheumatology response criterion 20 (ACR20) response at week 24. Secondary endpoints assessed as part of a predefined hierarchical hypothesis-testing strategy at week 24 included: proportion of patients with an ACR50 response; change from baseline in 28-joint Disease Activity Score based on C-reactive protein (DAS28-CRP); proportion of patients achieving Psoriasis Area Severity Index (PASI) 75 response in patients with psoriasis affecting ≥ 3% of body surface area; change from baseline in score of the Short Form-36 Physical Component Summary (SF-36 PCS); PASI 90 response; change from baseline in Health Assessment Questionnaire—Disability Index (HAQ-DI) score; and resolution of dactylitis and enthesitis.

Exploratory endpoints were: Functional Assessment of Chronic Illness Therapy—Fatigue (FACIT-Fatigue); change from baseline in patient’s assessment of PsA pain (visual analog scale (VAS)); assessments of all primary and secondary endpoints up to week 52; and subgroup analyses according to previous anti-TNF use (ACR20/50 and DAS28-CRP assessments) and concomitant MTX use (ACR20/50 assessments).

Autoinjector usability was assessed based on investigator/site staff rating scores of successful, hazard-free self-injection and patient rating of autoinjector acceptability. The Self-Injection Assessment Questionnaire (SIAQ) was used to evaluate patients’ perceptions before and after self-injection [20]. The SIAQ PRE module was completed by patients before the first self-injection during the baseline visit and assessed three domains (feelings about injections (FL), self-confidence (CO), and satisfaction with self-injection (SA)). The POST module included four domains (FL, CO, pain and skin reactions, and SA) at baseline and weeks 1 and 2.

Safety assessments included evaluation of all AEs and serious AEs (SAEs), together with electrocardiograms, physical examination, vital signs and laboratory assessments, and assessment of anti-secukinumab antibody development (immunogenicity).

### Statistical analysis

A sample size of 135 patients per group was estimated to provide about 98% power to detect a treatment difference for the primary endpoint of ACR20 response at week 24 assuming a placebo response of 21% and a secukinumab response of 47% based on Fisher’s exact test. Evaluations of efficacy were performed on the full analysis set (FAS), which comprised all randomized patients to whom treatment had been assigned. Closed testing procedures were used to maintain a family-wise error rate of 5% across the secukinumab groups and endpoints (details presented in Additional file 1).

Primary and other binary endpoints were evaluated by means of logistic regression, with treatment and anti-TNF response status as factors and weight as a covariate. Baseline PASI score was a covariate in PASI 75 and PASI 90 analyses. Missing values, including those due to discontinuation of study treatment, were imputed as failures to achieve the given response (nonresponses). Also, patients who did not achieve response based on joint count at week 16 were imputed as nonresponders at week 20 and week 24 (rescue penalty).

Between-group differences in continuous variables were evaluated with the use of a mixed-model repeated-measures (MMRM) approach, with missing data assumed to be missing at random, with treatment, assessment visit, and anti-TNF response status as factors. Weight and baseline values of endpoints were included in the model as continuous covariates, and treatment by analysis visit and baseline score by analysis visit as interaction terms. PASI 75/90 scores were also analyzed using a modified rescue penalty (Additional file 1). Subgroup analyses were carried out on the basis of previous anti-TNF therapy or concomitant MTX treatment.

SIAQ domain scores ranging from 0 to 10 were calculated to assess self-injection experience, with higher scores indicating a better experience for a patient. The percentage of patients who successfully completed self-injection per the instructions for use (IFU) was reported for the overall population at week 1 and SIAQ domain scores were provided by visit.

Safety endpoints were evaluated in the safety set, which included all patients who received at least one dose of the study drug; these endpoints were summarized descriptively. Safety results are presented for the placebo-controlled period and the entire safety-reporting period, which included all safety data up to the cutoff date of the last patient’s week 52 visit.

## Results

### Patients

**Fig. 1:**
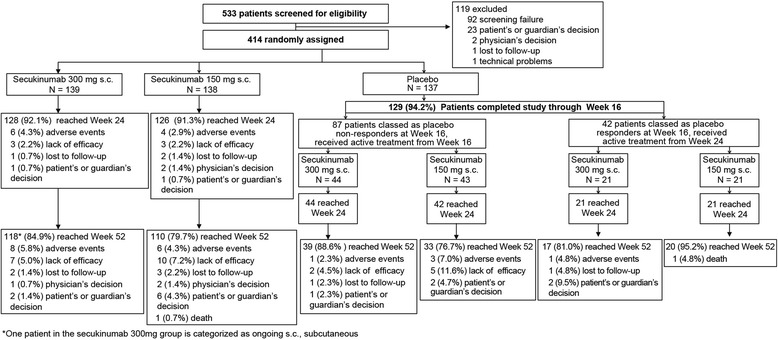
Patient disposition up to week 52

Overall, 414 patients with active PsA were enrolled in FUTURE 3. At baseline, demographic and disease characteristics were generally balanced across the treatment groups (Table 1). The mean time since the first diagnosis of PsA ranged from 6.6 to 8.3 years. Approximately two-thirds of the patients were anti-TNF-naïve (68.1%) and one-third (31.9%) were anti-TNF-IR, with patients in both subgroups evenly distributed across treatment groups; 53.8% of anti-TNF-IR patients were exposed to ≥ 2 anti-TNF agents. About half (47.6%) of the patients were using MTX at baseline (50.4%, secukinumab 300 mg; 42.8%, secukinumab 150 mg; 49.6%, placebo; Table 1). Of the 414 patients, 128/139 patients (92.1%, secukinumab 300 mg), 126/138 patients (91.3%, secukinumab 150 mg), and 128/137 patients (93.4%, placebo) completed 24 weeks and 337/414 patients (81.4%) completed 52 weeks (Fig. 1). The most common reasons for discontinuation by 52 weeks were lack of efficacy (5.0%, secukinumab 300 mg; 7.2%, secukinumab 150 mg) and AEs (5.8%, secukinumab 300 mg; 4.3%, secukinumab 150 mg). The most common reason for discontinuation in the initial placebo nonresponder group was lack of efficacy (4.5%, patients re-randomized to secukinumab 300 mg; 11.6%, patients re-randomized to secukinumab 150 mg).

**Table 1:** Demographic and baseline characteristics of patients

Characteristic	Secukinumab 300 mg (*N* = 139)	Secukinumab 150 mg (*N* = 138)	Placebo (*N* = 137)
Age (years), mean (SD)	49.3 (12.9)	50.1 (11.7)	50.1 (12.6)
Male, *n* (%)	67 (48.2)	61 (44.2)	59 (43.1)
Race, *n* (%)			
White	130 (93.5)	129 (93.5)	133 (97.1)
American Indian or Alaska Native	0	2 (1.4)	0
Asian	3 (2.2)	2 (1.4)	4 (2.9)
Other	6 (4.3)	5 (3.6)	0
Weight (kg), mean (SD)	87.1 (19.4)	87.1 (20.0)	82.6 (18.5)
Number of previous anti-TNF treatments for PsA, *n* (%)			
0	95 (68.3)	94 (68.1)	93 (67.9)
1	19 (13.7)	22 (15.9)	20 (14.6)
≥ 2	25 (18.0)	22 (15.9)	24 (17.5)
Time since diagnosis of PsA (years), mean (SD)	8.3 (9.2)	7.7 (8.5)	6.6 (6.9)
MTX use at randomization, *n* (%)	70 (50.4)	59 (42.8)	68 (49.6)
Systemic glucocorticoid use at randomization, *n* (%)	23 (16.5)	24 (17.4)	32 (23.4)
Anti-TNF-naïve, *n* (%)	95 (68.3)	94 (68.1)	93 (67.9)
Patients with specific disease characteristics, *n* (%)			
Psoriasis ≥ 3% of BSA	62 (44.6)	68 (49.3)	59 (43.1)
Presence of dactylitis	46 (33.1)	36 (26.1)	36 (26.3)
Presence of enthesitis	88 (63.3)	95 (68.8)	98 (71.5)
Disease and quality-of-life scores, mean (SD)			
TJC (78 joints)	19.7 (14.8)	23.3 (18.1)	21.9 (16.2)
SJC (76 joints)	8.9 (6.4)	11.2 (9.2)	10.3 (8.6)
DAS28-CRP	4.5 (1.0)	4.6 (1.1)	4.7 (1.1)
PASI^a^	10.1 (8.6)	8.8 (6.4)	10.4 (9.0)
Physician’s global assessment (VAS)	51.8 (19.7)	55.2 (16.7)	54.8 (18.1)
HAQ-DI	1.1 (0.7)	1.2 (0.6)	1.2 (0.6)
PsA pain (VAS)	54.8 (23.8)	54.4 (21.4)	53.3 (23.8)
Patient’s global assessment (VAS)	59.9 (20.8)	59.8 (22.1)	60.6 (20.9)
SF-36 PCS	39.2 (8.4)	37.9 (7.6)	37.4 (8.5)

*BSA* body surface area, *DAS28-CRP* 28-joint Disease Activity Score using C-reactive protein, *HAQ-DI* Health Assessment Questionnaire—Disability Index, *MTX* methotrexate, N number of randomized patients, *PASI* Psoriasis Area and Severity Index, *PsA* psoriatic arthritis, *SD* standard deviation, *SF-36 PCS* Short Form-36 Physical Component Summary, *SJC* swollen joint count, *TJC* tender joint count, *TNF* tumor necrosis factor, *VAS* visual analog scale

^a^Assessed in patients with psoriasis on at least 3% of their BSA

### Efficacy

**Fig. 2:**
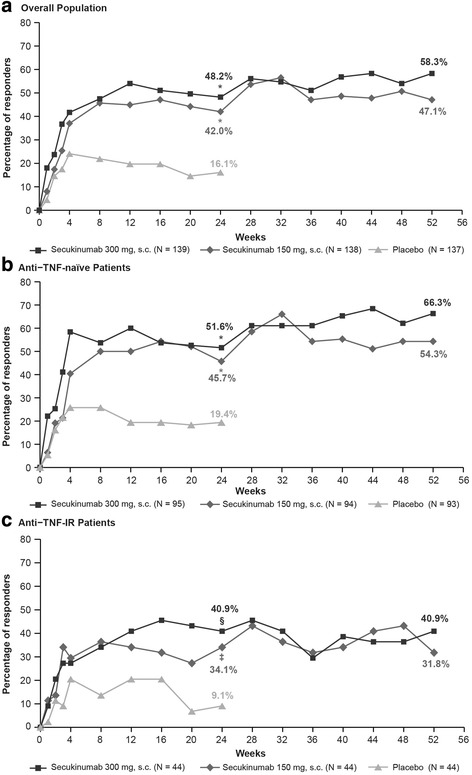
ACR20 response rates through week 52 in the overall population (**a**) and by anti-TNF status (**b, c**). ^*^p < 0.0001, ^§^*p* < 0.01, ^‡^*p* < 0.05 versus placebo. *p* values adjusted for multiplicity of testing for overall population at week 24. Missing values imputed as nonresponse (nonresponder imputation) through week 52. ACR20 20% improvement in American College of Rheumatology response criteria, IR inadequate response, s.c. subcutaneous, TNF tumor necrosis factor

**Fig. 3:**
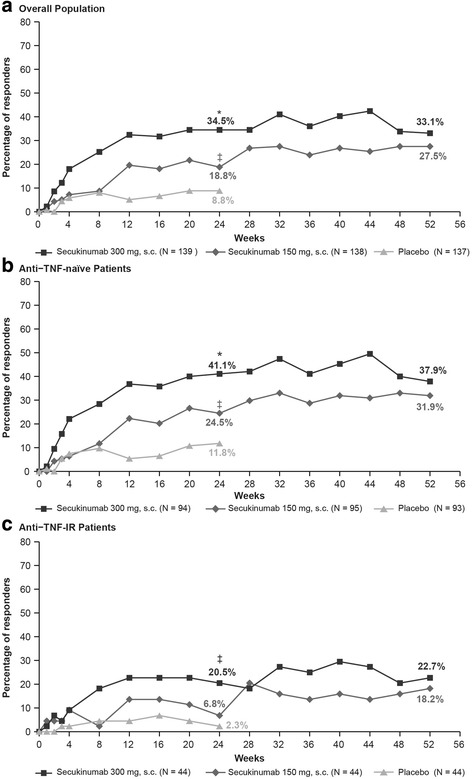
ACR50 response rates through week 52 in the overall population (**a**) and by anti-TNF status (**b, c**). ^*^p < 0.0001, ^‡^*p* < 0.05 versus placebo. *p* values adjusted for multiplicity of testing for overall population at week 24. Missing values imputed as nonresponse (nonresponder imputation) through week 52. ACR50 50% improvement in American College of Rheumatology response criteria, IR inadequate response, s.c. subcutaneous, TNF tumor necrosis factor

The primary endpoint was met, demonstrating efficacy of secukinumab (150 mg and 300 mg) versus placebo in the ACR20 response at week 24. ACR20 response rates using the conservative estimate of efficacy with missing values imputed as nonresponse were significantly higher in the secukinumab 300 mg (48.2%; *p* < 0.0001) and 150 mg (42.0%; *p* < 0.0001) groups versus placebo (16.1%; Fig. 2). Similarly, ACR50 response rates at week 24 were also significantly higher in the secukinumab 300 mg (34.5%; *p* < 0.0001) and 150 mg (18.8%; *p* < 0.05) groups versus placebo (8.8%; Fig. 3).

ACR20/50 response rates were higher with secukinumab than with placebo in both the anti-TNF-naïve and anti-TNF-IR patients, with response generally being higher in the anti-TNF-naïve population (Figs. 2 (ACR20) and 3 (ACR50)). At week 24, in anti-TNF-naïve patients the ACR20/50 response rates were 51.6%/41.1% (secukinumab 300 mg), 45.7%/24.5% (secukinumab 150 mg), and 19.4%/11.8% (placebo). In the anti-TNF-IR patients, the ACR20/50 response rates were 40.9%/20.5% in secukinumab 300 mg and 34.1%/6.8% in secukinumab 150 mg versus 9.1%/2.3% in the placebo group (Figs. 2 and 3).

ACR20/50 responses were sustained at week 52 in the overall population (58.3%/33.1% in secukinumab 300 mg group and 47.1%/27.5% in secukinumab 150 mg group). Response rates were also maintained in patients stratified by anti-TNF status through 52 weeks of treatment (Figs. 2 and 3).

Secondary endpoints were assessed in a hierarchical order. At week 24, statistical significance versus placebo was achieved for all secondary endpoints in the secukinumab 300 mg group. In the secukinumab 150 mg group, statistical significance was achieved for DAS28-CRP and PASI 75 scores. Clinical responses at week 24 across secondary endpoints with secukinumab 300 mg and 150 mg were sustained through 52 weeks of treatment (Table 2).

**Table 2:** Efficacy of secukinumab at weeks 24 and 52 in the overall population^a^

Endpoint	Week	Secukinumab 300 mg (*N* = 139)	Secukinumab 150 mg (*N* = 138)	Placebo (*N* = 137)
ACR20 response, *n*/*N* (%)	24	67/139 (48.2)^*^	58/138 (42.0)^*^	22/137 (16.1)
	52	81/139 (58.3)	65/138 (47.1)	–
ACR50 response, *n*/*N* (%)	24	48/139 (34.5)^*^	26/138 (18.8)^‡^	12/137 (8.8)
	52	46/139 (33.1)	38/138 (27.5)	–
DAS28-CRP, mean change from baseline ± SE	24	−1.56 ± 0.09^*^	−1.24 ± 0.1^‡^	−0.64 ± 0.13
	52	−1.61 ± 0.09	−1.41 ± 0.10	–
PASI 75 response, *n*/*N* (%)^b^	24	29/62 (46.8)^*^	34/68 (50.0)^‡^	6/59 (10.2)
	52	46/62 (74.2)	41/68 (60.3)	–
SF-36 PCS, mean change from baseline ± SE	24	6.46 ± 0.59^§^	3.42 ± 0.60	2.94 ± 0.83
	52	6.43 ± 0.66	4.49 ± 0.68	–
PASI 90 response, *n*/*N* (%)^b^	24	21/62 (33.9)^§^	25/68 (36.8)	4/59 (6.8)
	52	34/62 (54.8)	28/68 (41.2)	–
HAQ-DI score, mean change from baseline ± SE	24	− 0.38 ± 0.04^§^	− 0.27 ± 0.04	−0.17 ± 0.06
	52	−0.43 ± 0.05	−0.30 ± 0.05	–
Patients with resolution of dactylitis, *n*/*N* (%)^c^	24	22/46 (47.8)^§^	14/36 (38.9)	5/36 (13.9)
	52	28/46 (60.9)	19/36 (52.8)	–
Patients with resolution of enthesitis, *n*/*N* (%)^c^	24	35/88 (39.8)^§^	35/95 (36.8)	15/98 (15.3)
	52	47/88 (53.4)	44/95 (46.3)	–
Patient’s assessment of PsA pain (VAS), mean change from baseline ± SE	24	−18.23 ± 1.97^*^	−12.46 ± 2.0^‡^	−3.75 ± 2.81
	52	−20.3 ± 2.1	−11.8 ± 2.2	–
FACIT-Fatigue, mean change from baseline ± SE	24	6.40 ± 0.78^†^	2.73 ± 0.80	2.07 ± 1.05
	52	6.72 ± 0.9	3.25 ± 0.9	–

Data presented as nonresponder imputation and rescue penalty (binary variables) and mixed-model repeated measures and rescue penalty (continuous variables)

*ACR20/50* 20%/50% improvement in American College of Rheumatology response criteria; *BSA* body surface area, *DAS28-CRP* 28-joint Disease Activity Score using C-reactive protein, *FACIT-Fatigue* Functional Assessment of Chronic Illness Therapy—Fatigue, *HAQ-DI* Health Assessment Questionnaire—Disability Index, N number of patients, *PASI* Psoriasis Area Severity Index, *PsA* psoriatic arthritis, *SE* standard error, *SF-36 PCS* Short Form-36 Physical Component Summary, *VAS* visual analog scale

^*^p < 0.0001, ^†^*p* < 0.001, ^§^*p* < 0.01, ^‡^*p* < 0.05 versus placebo. *p* values adjusted for multiplicity of testing for overall population data (except for pain and FACIT-Fatigue)

^a^Prespecified primary and secondary endpoints were analyzed according to a statistical hierarchy except for pain and FACIT-Fatigue. Endpoints are shown in the order of testing

^**b**^PASI 75 and PASI 90 denote improvements of 75% and 90%, respectively, in the score on the Psoriasis Area Severity Index. Assessed in patients with psoriasis on at least 3% of their BSA

^**c**^Resolution of dactylitis and enthesitis among those patients with these symptoms at baseline: dactylitis, *N* = 46 (300 mg), 36 (150 mg), and 36 (placebo); enthesitis, *N* = 88 (300 mg), 95 (150 mg),and 98 (placebo)

Analysis of PASI 75/90 scores at week 24 using the modified rescue penalty (Additional file 1) resulted in 61.3%/41.9% and 55.9%/39.7% responses with secukinumab 300 and 150 mg, respectively, versus 11.9%/8.5% for placebo (*p* < 0.001).

In anti-TNF-naïve patients, mean change from baseline in the DAS28-CRP score at week 24 was −1.64 ± 0.10 (*p* < 0.0001) and −1.33 ± 0.10 (*p* < 0.01) in the secukinumab 300 and 150 mg groups, respectively, as compared with −0.78 ± 0.14 in the placebo group. In anti-TNF-IR patients, mean change from baseline in DAS28-CRP score was −1.43 ± 0.20 (*p* < 0.01) and −1.06 ± 0.19 (*p* > 0.05) in the secukinumab 300 and 150 mg groups, respectively, as compared with −0.49 ± 0.26 in the placebo group. The mean change from baseline in DAS28-CRP score observed with secukinumab at week 24 was sustained through week 52 regardless of anti-TNF status (anti-TNF-naïve, −1.68 ± 0.10 (secukinumab 300 mg) and −1.44 ± 0.11 (secukinumab 150 mg); anti-TNF-IR, −1.56 ± 0.21 (secukinumab 300 mg) and −1.47 ± 0.21 (secukinumab 150 mg)).

At week 24, ACR20/50 response rates were higher with secukinumab than with placebo in patients with and without concomitant MTX use, and the responses were maintained through 52 weeks of treatment (Additional file 1).

At week 24, mean change from baseline in patient’s assessment of PsA pain (VAS) was higher in both the secukinumab 300 and 150 mg groups versus placebo and mean change from baseline in FACIT-Fatigue was higher only in the secukinumab 300 mg group. The changes observed at week 24 in both exploratory endpoints were sustained through 52 weeks of treatment (Table 2).

Observed data at weeks 24 and 52 for the primary, secondary, and exploratory endpoints are presented in Additional file 1: Table S2.

### Autoinjector usability and satisfaction assessment

More than 99% of patients in the overall population successfully self-administrated the study drug at week 1. The most common hazard was needle stick in a noncritical area, observed in nine patients overall at randomization and week 1. The scores of FL, CO, and SA were high pre self-injection (FL = 8.22, CO = 6.52, and SA = 6.80) and remained high at week 2 (FL = 8.81, CO = 7.84, and SA = 8.39) in all patients. Patient-reported scores of the three domains by visit and treatment are shown in Additional file 1: Table S3. More than 90% of total patients reported no pain and reaction during or after the injection, and ≥ 88% of patients were either satisfied or very satisfied with the use of the autoinjector devise and found the usage of an autoinjector easy or very easy, with no external help required, as assessed by SIAQ at week 2.

### Safety

The incidence of AEs during the placebo-controlled period was similar across the study groups, with a slightly higher number of SAEs reported in the placebo group (Table 3). Treatment-emergent AEs were reported in 54.7%, 58.0%, and 56.2% of patients receiving secukinumab 300 mg, secukinumab 150 mg, and placebo, respectively. There were no deaths reported during the placebo-controlled 16-week period. Over the entire treatment period (mean ± SD exposure, secukinumab 398.1 ± 112.1 days and placebo 130.7 ± 29.8 days), exposure-adjusted incidence rates (EAIRs) of AEs among patients receiving secukinumab 300 and 150 mg were 194.9 and 192.5 per 100 patient-years, respectively; the corresponding values for SAEs were 8.8 and 10.2 per 100 patient-years, respectively (Table 3). The most frequent SAEs included infections and infestations and musculoskeletal and connective tissue disorders with an EAIR of 1.8 and 2.1, respectively. Details of SAEs through the entire safety period are presented in Additional file 1: Table S4. Two deaths were reported in the secukinumab 150 mg group during the entire 52-week analysis period: one pancreatic carcinoma and one small cell lung cancer (additional details regarding these patients are available in Additional file 1). Myocardial infarction was reported in one patient with a history of smoking and hyperlipidemia and who was a long-time NSAID user. Suicidal ideation was reported in two patients (one patient each in secukinumab 150 and 300 mg groups); both patients had a prior history of psychiatric disorders and were on related medications. *Candida* infections were reported for 14 patients in the secukinumab dose groups that included eight oral candidiasis (three patients in 300 mg group and five patients in 150 mg group) and four vulvovaginal candidiasis (two patients each in secukinumab 300 mg and secukinumab 150 mg groups). These *candida* infections were diagnosed as nonserious, mild, or moderate in severity by the investigators and did not lead to study withdrawal.

**Table 3:** Safety profile during the placebo-controlled period and the entire safety-reporting period

Variable	Through week 16 (placebo-controlled period)			Entire safety-data period		
	Secukinumab 300 mg (*N* = 139)	Secukinumab 150 mg (*N* = 138)	Placebo (*N* = 137)	Any secukinumab 300 mg (*N* = 204)	Any secukinumab 150 mg (*N* = 202)	Any secukinumab (*N* = 406)
Exposure to study treatment (days), mean ± SD				403.8 ± 108.3	392.3 ± 115.8	398.1 ± 112.1
Any AE	76 (54.7)	80 (58.0)	77 (56.2)	164 (194.9)	156 (192.5)	320 (193.7)
Any serious AE	3 (2.2)	5 (3.6)	9 (6.6)	19 (8.8)	21 (10.2)	40 (9.5)
Discontinued due to AEs^a^	3 (2.2)	5 (3.6)	5 (3.6)	9 (4.4)	13 (6.4)	22 (5.4)
Death^b^	0	0	0	0	2 (1.0)	2 (0.5)
Common AEs^c^						
Nasopharyngitis	13 (9.4)	11 (8.0)	13 (9.5)	47 (25.0)	30 (15.5)	77 (20.2)
Upper respiratory tract infection	7 (5.0)	6 (4.3)	5 (3.6)	23 (10.9)	19 (9.3)	42 (10.1)
Diarrhea	4 (2.9)	7 (5.1)	2 (1.5)	16 (7.5)	15 (7.2)	31 (7.4)
Back pain	5 (3.6)	4 (2.9)	1 (0.7)	16 (7.5)	12 (5.7)	28 (6.6)
Headache	6 (4.3)	9 (6.5)	6 (4.4)	11 (5.1)	15 (7.3)	26 (6.1)
Arthralgia	4 (2.9)	1 (0.7)	1 (0.7)	12 (5.5)	13 (6.3)	25 (5.9)
Bronchitis	3 (2.2)	2 (1.4)	5 (3.6)	15 (6.9)	9 (4.3)	24 (5.6)
Fatigue	5 (3.6)	4 (2.9)	2 (1.5)	7 (3.2)	15 (7.3)	22 (5.2)
Urinary tract infection	6 (4.3)	3 (2.2)	2 (1.5)	13 (6.0)	9 (4.2)	22 (5.1)
AEs of special interest						
Myocardial infarction	0	0	0	0	1 (0.5)	1 (0.2)
Neutropenia	0	0	0	1 (0.4)	2 (0.9)	3 (0.7)
*Candida* infection	0	2 (1.4)	2 (1.5)	6 (2.7)	8 (3.8)	14 (3.2)
IBD	0	0	1 (0.7)	1 (0.4)	0	1 (0.2)

Data are number (%) or number (incidence per 100 patient-years). In the analysis of the entire study period, the secukinumab groups include any patients who received the stated dose of secukinumab, including those who were randomly assigned to the placebo group at baseline and who underwent a second randomization to active treatment at week 16/24

*AE* adverse event, *IBD* inflammatory bowel disease, N number of patients, *SD* standard deviation

^a^Exposure-adjusted incidence rates were not calculated for discontinuations because of AEs and death

^b^Two deaths reported in the secukinumab 150 mg group: one due to pancreatic carcinoma and one due to small cell lung cancer on day 173 and day 227, respectively

^c^The most common AEs are reported as the preferred terms from the *Medical Dictionary for Regulatory Activities* and occurred at an incidence of at least 5 per 100 patient-years in the pooled secukinumab group during the entire treatment period

Malignant or unspecified tumors (excluding basal cell and squamous cell carcinomas) were reported in three patients each in the secukinumab 300 mg and 150 mg groups. One patient in the 300 mg group reported inflammatory bowel disease (moderate). Neutropenia was reported in three patients: one patient in the 300 mg group and two patients in the 150 mg group. This included Grade 3 leukopenia (one patient each in the 300 and 150 mg groups) and Grade 3 neutropenia (one patient in 150 mg group). Injection-site reactions were reported by six patients in the secukinumab 300 mg and seven patients in the secukinumab 150 mg group. Treatment-emergent anti-secukinumab antibodies (positive during study but negative at baseline) were detected in four patients, while neutralizing antibodies were not detected in any patients.

## Discussion

In this phase III trial, self-administration of secukinumab using an autoinjector in patients with PsA improved the signs and symptoms, skin measures, and physical function through 52 weeks of treatment. The ACR20/50 response rates with both secukinumab doses (300 and 150 mg) at week 24 were significantly higher than with placebo and were sustained through week 52. At week 24, significant outcomes with secukinumab 300 and 150 mg versus placebo were also observed in other clinically relevant domains of PsA, which were sustained through week 52. Patients were imputed as nonresponders based on joint count for all of the binary variables. These nonresponders included patients who were responding to variables with no/low correlation to joint assessment (e.g., PASI 75/90) at week 16. To overcome this, PASI 75/90 responses were also analyzed using a modified, variable-specific rescue penalty. The modified rescue penalty showed notably higher response rates for PASI 75 and PASI 90 compared to the original method in both secukinumab groups, with a minimal increase in the placebo group.

Results of this study are in agreement with the findings of earlier studies, which also showed significant improvements in the signs and symptoms of PsA with secukinumab 300 and 150 mg [6, 16, 18, 21]. Responses with secukinumab 150 mg observed in the present study, however, were lower than the responses observed in previous studies, with no obvious explanation other than interstudy variation.

In the subgroup analysis by previous anti-TNF use, clinical responses were observed with secukinumab in both anti-TNF-naïve and anti-TNF-IR populations, with responses generally greater in the anti-TNF-naïve subgroup. Substantial evidence suggests that treatment with anti-TNF agents reduces disease activity in patients with PsA [5–7]. However, some patients do not tolerate or respond well to these agents, and these refractory patients are commonly managed by switching from one anti-TNF to another. Glintborg et al. [22] reported that switching of anti-TNF agents in PsA patients was associated with decreasing ACR20 response rates from 47% with first-line anti-TNF therapy to 22% and 18% among patients switching to a second or third biologic, respectively. In this study, more than 50% of the patients in the anti-TNF-IR subgroup had received two or three anti-TNF agents and the ACR20 response rates at week 24 were 40.9% (secukinumab 300 mg) and 34.1% (secukinumab 150 mg). Higher responses observed with secukinumab 300 mg compared with 150 mg in patients previously exposed to anti-TNF agents are consistent with previous studies [6, 15, 16, 18] and provide additional supporting evidence that secukinumab 300 mg is an appropriate treatment option for this group of patients. Improvements with secukinumab through 52 weeks were similar in both concomitant MTX and without MTX subgroups.

This study showed the utility of the autoinjector device. More than 88% of patients were satisfied using the autoinjector and reported little or no pain at all. Moreover, the majority of the patients found usage of an autoinjector easy or very easy, with no external help required. These observations are in agreement with those recently reported for the secukinumab autoinjector in the psoriasis JUNCTURE trial [23].

The safety profile of secukinumab in this study was based on 398.1 patient-years of exposure to any secukinumab dose and showed no new or unexpected safety signals, and was consistent with earlier reports of secukinumab in PsA [16] and psoriasis [13]. The overall incidence of AEs up to week 16 was comparable between secukinumab dose groups and placebo. No dose dependence was observed for secukinumab. The majority of the AEs reported during the entire treatment period were nonserious and mild to moderate. All serious AEs in any secukinumab group for the entire treatment period were single events with no specific trends or clusters. The rate of discontinuations because of AEs with secukinumab was low, with two deaths reported during the study. *Candida* infections were reported more frequently with secukinumab (3.4%) than with placebo (1.5%); all were manageable with no treatment discontinuations. Since IL-17 plays a role in mucocutaneous defense against bacterial and fungal infections [24], continuous observation for *candida* infections is needed for these patients. Immunogenicity with secukinumab was low and was not associated with a loss of efficacy or with immunogenicity-related AEs.

This study has some limitations. This trial was not designed to identify a difference between doses. The long-term efficacy and safety of secukinumab compared to placebo could not be determined as patients on placebo had to be given rescue medication after a short period due to ethical consideration and study design, which was consistent with clinical trials of other biologic agents.

## Conclusions

In summary, self-administration of subcutaneous secukinumab provided sustained improvements at 52 weeks across multiple clinical domains in patients with active PsA. Patients reported high levels of satisfaction with the autoinjector and considered it easy to use and not painful. The safety profile of secukinumab showed no new or unexpected safety signals during this time.

## Additional file

Additional file 1: **Figure S1.** Showing study design, **Table S1.** Presenting independent ethics committees (IECs) or institutional review boards (IRB) by study center, **Table S2.** Presenting a summary of observed efficacy data at week 52 among patients randomized to secukinumab at baseline, **Table S3.** Presenting patient-reported acceptability of the autoinjector, and **Table S4.** Presenting SAEs by SOC reported across the entire study period. (DOCX 438 kb)

## Abbreviations

ACR: American College of Rheumatology
AE: Adverse event
CASPAR: ClASsification criteria for Psoriatic ARthritis
CO: Self-confidence
DAS-28 CRP: 28-joint Disease Activity Score based on C-reactive protein
DMARD: Disease-modifying anti-rheumatic drug
FACIT: Functional Assessment of Chronic Illness Therapy
FAS: Full analysis set
FL: Feelings about injections
HAQ-DI: Health Assessment Questionnaire—Disability Index
IEC: Independent ethics committee
IFU: Instructions for use
IL: Interleukin
IRB: Institutional review board
IRT: Interactive response technology
MMRM: Mixed-model repeated-measures
MTX: Methotrexate
NSAID: Nonsteroidal anti-inflammatory drug
PASI: Psoriasis Area Severity Index
PsA: Psoriatic arthritis
s.c.: Subcutaneous
SA: Satisfaction with self-injection
SF-36 PCS: Short Form-36 Physical Component Summary
SIAQ: Self-Injection Assessment Questionnaire
SJC: Swollen joint count
TJC: Tender joint count
TNF: Tumor necrosis factor
TNF-IR: Tumor necrosis factor inhibitor inadequate responder
VAS: Visual analog scale
