# Supplementary material for: Efficacy and safety of secukinumab administration by autoinjector in patients with psoriatic arthritis: results from a randomized, placebo-controlled trial (FUTURE 3)
Source: Arthritis Res Ther. 2018 Mar 15;20:47. doi: 10.1186/s13075-018-1551-x (PMC5856314; doi:10.1186/s13075-018-1551-x)
Supplement: Supplementary file 1 — Figure S1. Showing study design, Table S1. Presenting independent ethics committees (IECs) or institutional review boards (IRB) by study center, Table S2. Presenting a summary of observed efficacy data at week 52 among patients randomized to secukinumab at baseline, Table S3. Presenting patient-reported acceptability of the autoinjector, and Table S4. Presenting SAEs by SOC reported across the entire study period. (DOCX 438 kb) [file 13075_2018_1551_MOESM1_ESM.docx]

**Additional file 1**

**MATERIALS AND METHODS**

**Figure S1. Study design**


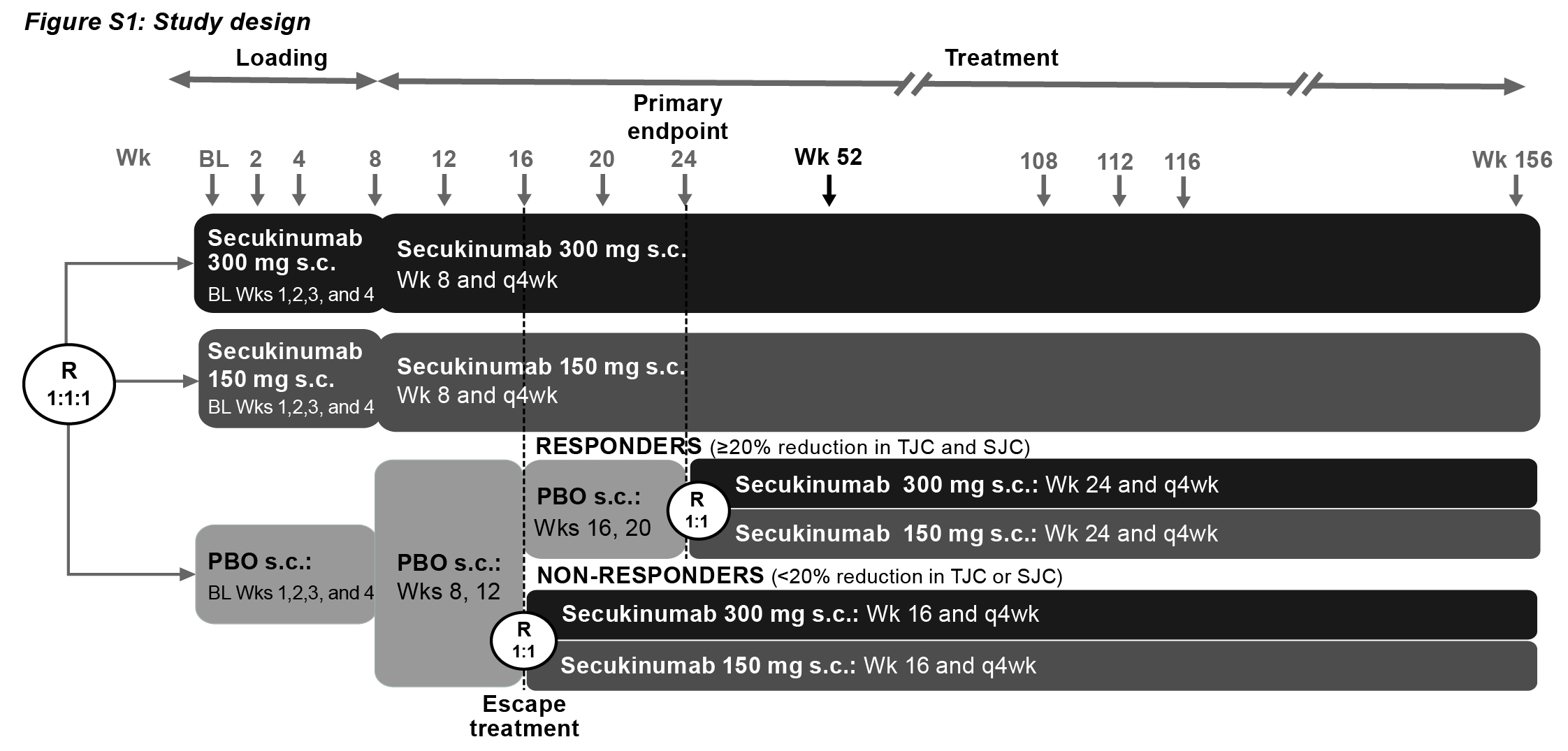


BL: baseline; PBO: placebo; q4wk: every 4 weeks; R: randomization; s.c.: secukinumab; SJC: swollen joint count; TJC: tender joint count; Wk: week.

**Statistical analysis**

**Details of the pre-specified hierarchy**

The hypotheses for the primary objective in either the secukinumab treatment arm versus placebo were tested simultaneously at the p=0.025 level. Based on the rejection of 1 or both hypotheses, secondary endpoint analysis was completed according to the following pre-specified hierarchy sequence at p=0.025 level: ACR50, DAS28-CRP, PASI 75, SF-36 PCS, PASI 90, HAQ-DI, dactylitis, and enthesitis. . If all secondary endpoints were rejected for one of the secukinumab arms vs. placebo the testing strategy allowed for its assigned alpha of 2.5% to be transferred to the testing sequence for the other arm. Hence, the hypotheses for that secukinumab arm vs. placebo could then be tested at 5% level (if not already rejected at 2.5% level).

**Modified rescue penalty**

For the primary analyses, all patients who were non-responders based on the joint count at Week 16 were imputed as non-responders at Week 20 and Week 24 for all binary variables (rescue penalty). This was done also for secukinumab patients although they continued on the same dose of secukinumab. A consequence of this approach was that patients who were responding to variables with no or low correlation to joint assessment (e.g., PASI 75) at Week 16 were considered non-responders at Week 24 because they did not demonstrate improvements in joints. Therefore, a new variable-specific penalty method was introduced for exploratory purposes where patients also had to be Week 16 non‑responders for the specific variable under study to have rescue applied.

**RESULTS**

**ACR20/50 responses in patients with and without concomitant MTX use**

At Week 24, ACR20/50 response rates were higher with secukinumab than with placebo in patients with and without concomitant MTX use; concomitant MTX: ACR20/50 was 42.9%/30.0% in secukinumab 300mg (p<0.01 for ACR20 and p<0.01 for ACR50), 50.8%/27.1% in secukinumab 150mg (p<0.001 for ACR20 and p<0.01 for ACR50) groups versus 20.6%/10.3% in placebo group; without concomitant MTX: ACR20/50 was 53.6%/39.1% in secukinumab 300mg (p<0.0001 for ACR20 and p<0.0001 for ACR50) and 35.4%/12.7% in secukinumab 150mg groups (p<0.01 for ACR20 and p>0.05 for ACR50) versus 11.6%/7.2% in placebo group. These responses were maintained through 52 weeks of treatment.

**Safety**

**Details of death**

Two deaths were reported in the secukinumab 150mg group during the entire treatment period, 1 due to pancreatic carcinoma (considered not related to the study treatment) and the other due to small cell lung cancer (considered related to the study treatment). The small cell lung cancer patient had a history of cigarette smoking and obesity. Treatment with the study medication was permanently discontinued due to the events (small cell lung cancer metastatic, metastases to the spine, liver, and central nervous system), with the last dose reported on Day 196. The patient died on Day 240 due to the above described events.

**Table S1. List of independent ethics committees (IEC) or Institutional Review Boards (IRB) by study center**

| **Centre Numbers** | **Centre Name** | **Ethics Committee or Institutional Review Board** | **City, Country** | **Institutional Review Board Approval/Reference Numbers/dates** |
| --- | --- | --- | --- | --- |
| 1001 |  | CEIC Hospital Clinic de Barcelona | Villarroel, 170 Barcelona 08036 | HCP/2013/251 |
| 1003 |  | Comité Etico de Investigacion Clínica de Cantabria | Unidad: IDIVAL Instituto de Investigación Marqués de Valdecilla  Dirección: Edificio IFIMAV 3ª Planta  Avda Cardenal Herrera Oria s/n Santander 39011 | HCP/2013/251 |
| 1005 |  | Comité Autonómico de Ética de la Investigación de Galicia | Unidad: Secretaria Xeral. Conselleria de Sanidade  Dirección: C/ San Lázaro, s/n  Santiago de Compostela  15781 | HCP/2013/251 |
| 2001 | Optimus Clinical Research | The Bellberry HREC | Eastwood SA 5063 Australia | 2014-01-712 |
| 2002 | Emeritus Research | The Bellberry HREC | Eastwood SA 5063 Australia | 2014-01-712 |
| 2003 | Southern Clinical Research | Tasmania Health and Medical Human Research Ethics Committee, University of Tasmania Churchill Avenue | Sandy Bay TAS 7005 Australia | H0013891 |
| 2004 | Rheumatology Research Unit | The Bellberry HREC | Eastwood SA 5063 Australia | 2014-01-712 |
| 3001 | Military Medical Academy - MHAT Sofia, Department of rheumatology | Ethics Committee for Multicenter Trials | Sofia 1000 Bulgaria | KI-53/21.05.14 |
| 3002 | University Hospital for Active Treatment "Sv. Ivan Rilski" EAD Sofia, Rheumatology clinic | Ethics Committee for Multicenter Trials | Sofia 1000 Bulgaria | KI-53/21.05.14 |
| 3003 | Diagnostic-Consultative Center XVII - Sofia EOOD, Rheumatology office | Ethics Committee for Multicenter Trials | Sofia 1000 Bulgaria | KI-53/21.05.14 |
| 3004 | Multiprofile District Hospital for Active Treatment "Dr. Stefan Cherkezov" AD, Veliko Tarnovo, Second department of Internal diseases | Ethics Committee for Multicenter Trials | Sofia 1000 Bulgaria | KI-53/21.05.14 |
| 3501 |  | IRB Services | Aurora Ontario L4G 0A5 Canada | v3, 2014-JAN-22 |
| 3502 |  | IRB Services | Aurora Ontario L4G 0A5 Canada | v3, 2014-JAN-22 |
| 3503 |  | IRB Services | Aurora Ontario L4G 0A5 Canada | v3, 2014-JAN-22 |
| 3504 |  | IRB Services | Aurora Ontario L4G 0A5 Canada | v3, 2014-JAN-22 |
| 4001 | Revmatologie s.r.o, Zahradní 979/16, 792 01 Bruntál | Local IEC: Etická komise, Slezská nemocnice p.o. | Opava 746 01 Czech republic | 148/2014 EK SON from 02-Apr-2014 |
| 4002 | Medical Plus s.r.o, Obchodní 1507, 686 01 Uherské Hradiště | Local IEC: Etická komise Uherskohradiststké nemocnice | Uherské Hradiště 686 68 Czech republic | Decision from 31-Mar-2014 (approval number NA) |
| 4003 | Revmatologický ústav, Na Slupi 4, 128 50 | Local IEC: Etická komise Revmatologického ústavu | Prague2 128 50 Czech republic | 1557/2014 from 25-Mar-2014 |
| Czech Republic | National Ethics Committee | Etická komise IKEM a FTN | Vídeňská 800, 140 59 Praha 4 - Krč | 418/2014 (M-14-15) from 12-Mar-2014 |
| 4503 | Prof. Burmester  Charité Campus Mitte Universitätsmedizin Berlin  Med. Klinik mit Schwerpunkt Rheumatologie und Klinische Immunologie | Landesamt für Gesundheit und Soziales Ethik-Kommission des Landes Berlin | Berlin 10707 Germany | Ethik-Kommission 14017 |
| 4504 | Dr. Dahmen  Praxis für klinische Studien | Ärztekammer Hamburg Geschäftsstelle der Ethik- Kommission | Hamburg 22083 Germany | Ethik-Kommission 14017 |
| 4505 | Dr. Demary  Praxis | Ärztekammer Niedersachsen Ethik-Kommission | Hannover 30175 Germany | Ethik-Kommission 14017 |
| 4506 | Dr. Everding  HRF Hamburger Rheuma Forschungszentrum II, MVZ Rheumatologie und Autoimmunmedizin Hamburg GmbH, Dr. Andrea Everding GbR | Ärztekammer Hamburg Geschäftsstelle der Ethik- Kommission | Hamburg 22083 Germany | Ethik-Kommission 14017 |
| 4507 | Prof. Kekow  Immunologisches Zentrum Vogelsang-Gommern GmbH | Landesamt für Verbraucherschutz Ethik-Kommission des Landes Sachsen-Anhalt | Dessau-Rosslau 06846 Germany | Ethik-Kommission 14017 |
| 4508 | Dr. Kurthen  Praxis | Ärztekammer Nordrhein Ethik-Kommission | Düsseldorf 40474 Germany | Ethik-Kommission 14017 |
| 4509 | Dr. Lorenz  MVZ AGILOMED | Sächsische Landesärztekammer Ethik-Kommission | Dresden 01099 Germany | Ethik-Kommission 14017 |
| 4510 | Prof. Möricke  Institut für Präventive Medizin & Klinische Forschung GbR | Landesamt für Verbraucherschutz Ethik-Kommission des Landes Sachsen-Anhalt | Dessau-Rosslau 06846 Germany | Ethik-Kommission 14017 |
| 4512 | Prof. Schmidt  Medizinische Hochschule Hannover, Zentrum Innere Medizin, Klinik für Immunologie und Rheumatologie | Ethik-Kommission der Medizinischen Hochschule Hannover | Hannover 30625 Germany | Ethik-Kommission 14017 |
| 4513 | Dr. Schuch  Gemeinschaftspraxis | Ethik-Kommission der Bayerischen Landesärztekammer | München 81677 Germany | Ethik-Kommission 14017 |
| 4515 | Dr. Spieler  ZEFOR GmbH, Zentrum f. Forschung Osteologie u. Rheumatologie | Landesamt für Verbraucherschutz Ethik-Kommission des Landes Sachsen-Anhalt | Dessau-Rosslau 06846 Germany | Ethik-Kommission 14017 |
| 4517 | Prof. Wollenhaupt  Schön Klinik Hamburg-Eilbek | Ärztekammer Hamburg Geschäftsstelle der Ethik-Kommission | Hamburg 22083 Germany | Ethik-Kommission 14017 |
| 5001 | Elizabeth Barranco Ponce School Of Medicine | Quorum | Seattle WA 98101 USA | QR#: 29005/7 |
| 5003 | John Budd Clayton Medical Research | Quorum | Seattle WA 98101 USA | QR#: 29005/5 |
| 5004 | Tina Bunch Austin Regional Clinic | Quorum | Seattle WA 98101 USA | QR#: 29005/11 |
| 5005 | James Byrd Wenatchee Valley Hospital & Clinics | Quorum | Seattle WA 98101 USA | QR#: 29005/10 |
| 5006 | Hisham El-Kadi Arthritis and Osteoporosis Associates | Quorum | Seattle WA 98101 USA | QR#: 29005/8 |
| 5008 | Scott Fretzin DAWES-FRETZIN Clinical Research | Quorum | Seattle WA 98101 USA | QR#: 29005/17 |
| 5010 | Jeffrey Kaine Sarasota Arthritis Research Center | Quorum | Seattle WA 98101 USA | QR#: 29005/19 |
| 5012 | Alan Kivitz Altoona Center for Clinical Research | Quorum | Seattle WA 98101 USA | QR#: 29005/3 |
| 5015 | Joel Kremer The Center for Rheumatology, LLP | Quorum | Seattle WA 98101 USA | QR#: 29005/2 |
| 5018 | Karen Zager Arthritis Center | Quorum | Seattle WA 98101 USA | QR#: 29005/12 |
| 5023 | Atul Singhal SOUTHWEST RHEUMATOLOGY | Quorum | Seattle WA 98101 USA | QR#: 29005/4 |
| 5029 | Jose Rabelo Centro de Investigacion Clinica | Quorum | Seattle WA 98101 USA | QR#: 29005/18 |
| 5030 | Norman Gaylis Arthritis and Rheumatic Disease Specialties | Quorum | Seattle WA 98101 USA | QR#: 29005/16 |
| 6001 |  | Comitato etico dell'irccs | Rozzano MI 20089 Italy |  |
| 6003 |  | Comitato etico per la sperimentazione clinica delle province di verona e rovigo presso aoui verona | Verona VR 37134 Italy |  |
| 6004 |  | Comitato etico indipendente dell'azienda ospedalierouniversitaria policlinico s.orsola - malpighi di bologna | Bologna BO 40138 Italy |  |
| 6005 |  | Comitato etico interaziendale a.o. citta' della salute e della scienza di torino | Torino to 10126 Italy |  |
| 6006 |  | Comitato etico catania 1 | Catania CT 95123 Italy | Ministerial Decree of 21/12/2007 |
| 6008 |  | Comitato Etico Regione Liguria c/o Irccs Aou San Martino | Genova GE 16132 Italy |  |
|  | Zuyderland Ziekenhuis, Heerlen | St. Antonius Ziekenhuis, MEC-U | Nieuwegein 3430 EM, Netherlands | M13-1385 / M13-085 |
| 6502 | Academisch Medisch Centrum, Amsterdam | St. Antonius Ziekenhuis, MEC-U | Nieuwegein 3430 EM, Netherlands | M13-1385 / M13-085 |
| 6504 | Maasstad ziekenhuis, Rotterdam | St. Antonius Ziekenhuis, MEC-U | Nieuwegein 3430 EM, Netherlands | M13-1385 / M13-085 |
| 7501 | Prof O. Ershova  Yaroslavl Soloviyev Clinical Hospital (State authonomic clinical hospital for Emergency Medical Care n.a.N.V.Solovyev of Yaroslavl region) | Ethic Committee of Soloviyev Clinical Hospital | Yaroslavl 150003 Russia | EC Meeting # 44 date 08 May 2014 |
| 7502 | Prof. O.Nesmeyanova  Chelyabinsk Region Clinical Hospital (State Budget Institution of Health Chelyabinsk Regional Hospital) | Ethic Committee of Chelyabinsk Region Clinical Hospital | Chelyabinsk 454076 Russia | EC meeting #11 date 10 July 2014 |
| 7504 | Prof . A.Kastanayan  Rostov-on-Don State Med.University (Federal State Budgetary Educational Institution of Higher Education «The Rostov State Medical University» of Ministry of Health of the Russian Federation) | Ethic Committee under Rostov on Don State Medical University | Rostov on Done 344022 Russia | EC Meeting 9/14 date 15 May 2014 |
| 7505 | Prof .A Rebrov  Saratov State Medical University (State Healthcare Institution "Regional Clinical Hospital") | LEC of Saratov Regional Clinical Hospita | Saratov 410053 Russia | EC meeting #14-03 date 30 Apr 2014 |
| 7506 | Dr.M.Stanislav  Rheumatology Inst. of Russian Academy of Medical Sciences (Federal state budgetary institution "Research Institute of Rheumatology named after V.A. Nasonova") | Ethic Committee under Research Institute of Rheumatology | Moscow 115522 Russia | EC meeting # 14 date 08 May 2014 |
| 7507 | Dr.A.Agafina  Municipal hospital # 40 of the Kurortnyi Region (Saint-Petersburg State Healthcare Institution "City Hospital # 40 of the Resort Administrative District") | LEC of city hospital # 40 | Sestroreck , St-Petersburg 197706 Russia | EC meeting #68 date 30 Jun 2014 |
| 7509 | Prof. N.Izmozherova  Ural State Medical Academy (Federal State Budgetary Educational Institution of Higher Education "Ural State Medical University" of Ministry of Health of Russian Federation) | Ethic Committee in Ural State medical Academy | Ekaterinburg 620219 Russia | EC meeting # 6 date 20 Jun 2014 |
| 8501 | Kantonsspital St. Gallen  Rheumatologie | Ethikkommission des Kantons St. Gallen | St. Gallen 9007 Switzerland | Ref. 14/062/L |
| 8502 | Infirmière de recherche clinique  Service de Rhumatologie  Hôpital cantonal de Fribourg | Commission cantonale d'éthique de la recherche sur l'être humain | Lausanne 1012 Switzerland | Ref. 218/14 |
| 9001 |  | NRES Committee East Midlands –Leicester | Nottingham NG1 6FS United Kingdom | 14/EM/0024 |
| 9002 |  | NRES Committee East Midlands –Leicester | Nottingham NG1 6FS United Kingdom | 14/EM/0024 |
| 9003 |  | NRES Committee East Midlands –Leicester | Nottingham NG1 6FS United Kingdom | 14/EM/0024 |
| 9004 |  | NRES Committee East Midlands –Leicester | Nottingham NG1 6FS United Kingdom | 14/EM/0024 |
| 9004 |  | NRES Committee East Midlands –Leicester | Nottingham NG1 6FS United Kingdom | 14/EM/0024 |
| 9005 |  | NRES Committee East Midlands –Leicester | Nottingham NG1 6FS United Kingdom | 14/EM/0024 |
| 9006 |  | NRES Committee East Midlands –Leicester | Nottingham NG1 6FS United Kingdom | 14/EM/0024 |
| 9007 |  | NRES Committee East Midlands –Leicester | Nottingham NG1 6FS United Kingdom | 14/EM/0024 |
| 9008 |  | NRES Committee East Midlands –Leicester | Nottingham NG1 6FS United Kingdom | 14/EM/0024 |
| 9009 |  | NRES Committee East Midlands –Leicester | Nottingham NG1 6FS United Kingdom | 14/EM/0024 |
| 9010 |  | NRES Committee East Midlands –Leicester | Nottingham NG1 6FS United Kingdom | 14/EM/0024 |
| 9011 |  | NRES Committee East Midlands –Leicester | Nottingham NG1 6FS United Kingdom | 14/EM/0024 |
| 9012 |  | NRES Committee East Midlands –Leicester | Nottingham NG1 6FS United Kingdom | 14/EM/0024 |
| 9013 |  | NRES Committee East Midlands –Leicester | Nottingham NG1 6FS United Kingdom | 14/EM/0024 |

**Table S2. Summary of observed efficacy data at Week 52 among patients randomized to secukinumab at baseline**

| **Efficacy endpoint** | **Secukinumab**  **300mg, s.c.** | **Secukinumab**  **150mg, s.c.** |
| --- | --- | --- |
| **ACR20 response, n/N (%)** | 81/118 (68.6) | 65/107 (60.7) |
| **ACR50 response, n/N (%)** | 46/118 (39.0) | 38/107 (35.5) |
| **DAS28-CRP, mean change from baseline (SD)** | −1.60 (1.12) | −1.61 (1.15) |
| **PASI 75 response,** **n/N (%)^#^** | 46/54 (85.2) | 41/57 (71.9) |
| **SF-36 PCS, mean change from baseline (SD)** | 6.27 (8.09) | 5.50 (7.13) |
| **PASI 90 response,** **n/N (%)^#^** | 34/54 (63.0) | 28/57 (49.1) |
| **HAQ-DI score, mean change from baseline (SD)** | −0.42 (0.57) | −0.40 (0.56) |
| **Absence of dactylitis,** **n/N (%)** | 106/118 (89.8) | 98/111 (88.3) |
| **Absence of enthesitis, n/N (%)** | 89/118 (75.4) | 78/111 (70.3) |
| **Patient’s assessment of PsA pain (VAS), mean change from baseline (SD)** | −22.2 (28.2) | −14.7 (27.4) |
| **FACIT-Fatigue, mean change from baseline (SD)** | 6.64 (10.4) | 4.03 (10.3) |
| **^#^**PASI 75 and PASI 90 denote improvements of 75% and 90%, respectively, in the score on the Psoriasis Area Severity Index. Assessed in patients with psoriasis on at least 3% of their BSA.  ACR: American College of Rheumatology response criteria; BSA: body surface area; DAS28‑CRP: 28-joint Disease Activity Score including levels of C-reactive protein; FACIT‑Fatigue: Functional Assessment of Chronic Illness Therapy–Fatigue; HAQ-DI: Health Assessment Questionnaire–Disability Index; n: number of patients who are responders; N: total number of patients in the treatment group with evaluation; PASI: Psoriasis Area Severity Index; PsA: psoriatic arthritis; s.c.: subcutaneous; SD: standard deviation; SF-36 PCS: Short Form-36 Physical Component Summary; VAS: visual analog scale. | | |

**Table S3. Patient-reported acceptability of the autoinjector.**

|  | **Baseline (PRE module)** | | | **Week 2 (POST module)** | | |
| --- | --- | --- | --- | --- | --- | --- |
| **Domain scores,**  **mean (SD)** | **Secukinumab**  **300mg, s.c.**  **(N = 139)** | **Secukinumab**  **150mg, s.c.**  **(N = 138)** | **Placebo**  **(N = 137)** | **Secukinumab**  **300mg, s.c.**  **(N = 139)** | **Secukinumab**  **150mg, s.c.**  **(N = 138)** | **Placebo**  **(N = 137)** |
| FL | 8.3 (1.8) | 8.4 (2.0) | 8.0 (2.2) | 9.0 (1.8) | 8.7 (2.0) | 8.8 (1.6) |
| CO | 6.6 (2.7) | 6.6 (2.7) | 6.4 (2.8) | 8.0 (2.2) | 7.7 (2.9) | 7.9 (2.2) |
| SA | 6.6 (2.4) | 6.9 (2.4) | 6.9 (2.3) | 8.4 (1.8) | 8.2 (2.0) | 8.6 (1.5) |
| Overall patient experience with secukinumab administration via the autoinjector was assessed at baseline (PRE module) and at Week 2 (POST module) by SIAQ domains  CO: self-confidence; FL: feelings about injections; N: number of randomized patients; SA: satisfaction with self-injection; s.c.: subcutaneous; SD: standard deviation; SIAQ: self-injection assessment questionnaire. | | | | | | |

**Table S4. Serious AEs by SOC reported across the entire study period**

| **Serious AE by SOC,** | **Any^a^ secukinumab 300mg, s.c.**  **(N = 204)**  **n (EAIR)** | **Any^a^ secukinumab 150mg, s.c.**  **(N = 202)**  **n (EAIR)** | **Any^a^  secukinumab**  **(N = 406)**  **n (EAIR)** |
| --- | --- | --- | --- |
| **Cardiac disorders** | 1 (0.4) | 2 (0.9) | 3 (0.7) |
| **Ear and labyrinth disorders** | 0 | 1 (0.5) | 1 (0.2) |
| **Eye disorders** | 0 | 2 (0.9) | 2 (0.5) |
| **Gastrointestinal disorders** | 1 (0.4) | 2 (0.9) | 3 (0.7) |
| **General disorders and administration site conditions** | 0 | 3 (1.4) | 3 (0.7) |
| **Hepatobiliary disorders** | 0 | 1 (0.5) | 1 (0.2) |
| **Infections and infestations** | 5 (2.2) | 3 (1.4) | 8 (1.8) |
| **Injury, poisoning, and procedural complications** | 4 (1.8) | 1 (0.5) | 5 (1.1) |
| **Metabolism and nutrition disorders** | 2 (0.9) | 1 (0.5) | 3 (0.7) |
| **Musculoskeletal and connective tissue disorders** | 3 (1.3) | 6 (2.8) | 9 (2.1) |
| **Neoplasms benign, malignant, and unspecified (incl cysts and polyps)** | 4 (1.8) | 3 (1.4) | 7 (1.6) |
| **Nervous system disorders** | 0 | 1 (0.5) | 1 (0.2) |
| **Psychiatric disorders** | 1 (0.4) | 1 (0.5) | 2 (0.5) |
| **Reproductive system and breast disorders** | 1 (0.4) | 0 | 1 (0.2) |
| **Skin and subcutaneous tissue disorders** | 0 | 1 (0.5) | 1 (0.2) |
| **Vascular disorders** | 1 (0.4) | 2 (0.9) | 3 (0.7) |
| AE: adverse event; EAIR: exposure-adjusted incidence rate; s.c.: subcutaneous; SOC: system organ class.  ^a^The secukinumab groups in this period include any patients who received the stated dose of secukinumab and include those patients randomized to placebo at baseline who were re-randomized to active treatment from Week 16 or Week 24 depending upon the clinical response. | | | |
